# Influence of surgical position and registration methods on clinical accuracy of navigation systems in brain tumor surgery

**DOI:** 10.1038/s41598-023-29710-w

**Published:** 2023-02-14

**Authors:** Motomasa Furuse, Naokado Ikeda, Shinji Kawabata, Yangtae Park, Koji Takeuchi, Masao Fukumura, Yuichiro Tsuji, Seigo Kimura, Takuya Kanemitsu, Ryokichi Yagi, Naosuke Nonoguchi, Toshihiko Kuroiwa, Masahiko Wanibuchi

**Affiliations:** Department of Neurosurgery, Osaka Medical and Pharmaceutical University, Takatsuki, Osaka Japan

**Keywords:** Neurological disorders, Surgery

## Abstract

The aim of this study was to evaluate the influence of skin distortion due to surgical positioning on the clinical accuracy of the navigation system. The distance errors were measured in four fiducial markers (anterior, posterior, right, and left of the head) after the registration of the navigation system. The distance errors were compared between the surface-merge registration (SMR) method using preoperative imaging and the automatic intraoperative registration (AIR) method using intraoperative imaging. The comparison of the distance errors were performed in various surgical positions. The AIR method had the significant accuracy in the lateral markers than the SMR method (lateral position, 3.8 mm vs. 8.95 mm; *p *< 0.0001; prone position, 4.5 mm vs. 13.9 mm; *p *= 0.0001; 5.2 mm vs. 11.5 mm; *p *= 0.0070). The smallest distance errors were obtained close to the surgical field in the AIR method (3.25–3.85 mm) and in the forehead in the SMR method (3.3–8.1 mm). The AIR method was accurate and recommended for all the surgical positions if intraoperative imaging was available. The SMR method was only recommended for the supine position, because skin distortion was frequently observed in the lateral region.

## Introduction

The development of operative assistance equipment, including neuromonitoring, neuroendoscopy, photodiagnosis, surgical navigation systems, and intraoperative imaging, has contributed to advances in brain tumor surgery over the past few decades. Modern brain tumor surgery aims to safely perform maximal resection, using accurate anatomical and functional information. As we know that navigation systems use preoperative images to create a similar image in space that corresponds to that in the patient. Registration is the process of matching a patient’s spatial data with the spatial radiological imaging, and is the most important procedure for the accuracy of the navigation system. Generally, two image-to-patient registration techniques have been developed: point-based registration and iterative closest point registration^1^. Initially, the point-based registration used fiducial markers implanted on the skin surface. Information of specified positions of the paired points were decided by surgeons and fed into the system for calculation of the transformation matrix. Then the coordinates of the registration points on patient’s body were obtained using an optical navigation probe^2^. Subsequently, the iterative closest point method, which uses surface matching of anatomical information instead of implanted fiducial markers, has been widely adopted^1^. A large number of points on the skin obtained by surface tracing were digitized. Random points on the skin surface were matched, and coordinate transformation of these points were calculated^3^. This surface-merge registration (SMR) using the iterative closest point method is a cost-effective technique that may reduce patient burden because it is not necessary to perform additional preoperative imaging with implanted fiducial markers. Although SMR is practical, its accuracy is inferior to that of point-based registration^4^.

Intraoperative imaging has been applied as a reference to the imaging space in navigation systems. This may improve the accuracy of the navigation system, as intraoperative imaging is performed in patients who have been placed in the surgical position with the head fixed. Preoperative imaging, which has been performed in a position different from the patient’s surgical position, may cause distance errors in the navigation system owing to skin distortion. Moreover, navigation systems can be automatically registered using intraoperative images without traces of the surface anatomy. However, intraoperative imaging is not commonly used, and registration using preoperative imaging is frequently adopted instead. The clinical accuracy of the navigation system, which was obtained in operating room, was different from the nominal accuracy, which was measured in lab environment. The clinical accuracy was influenced by lack of line-of -sight for the navigation system, skin distortion due to loss of muscle tone, intubation, nasogastric tube placement, patient positioning, and skin movement due to use of the navigation pointer^5^. We hypothesized that the automatic intraoperative registration (AIR) method, using intraoperative imaging as a reference, would be accurate and could be used as a control to evaluate the clinical accuracy of the SMR method in various surgical positions. Therefore, we measured and compared the distance errors of the navigation system between the SMR method using preoperative imaging and the AIR method to elucidate the skin distortion against the whole head. Herein, we aimed to evaluate the influence of skin distortion according to surgical position on the clinical accuracy of the navigation system.

## Methods

This study was approved by the institutional ethics committee of Osaka Medical and Pharmaceutical University (No. R332 1975-01, UMIN000023263, |July 20th, 2018 |). The study was carried out in accordance with the ethical standards of the responsible committee on institutional human experimentation and with Helsinki Declaration of 1975. Written informed consent was obtained from all patients. This was a single-arm prospective study. Patients were eligible if they were ≥ 20 years old and had a tolerable physical status for general anesthesia and surgery. Patients scheduled for brain tumor surgery who were planned to undergo intraoperative computed tomography (CT) were enrolled in this study. Distance error was measured in terms of the clinical accuracy of the navigation system and compared between the AIR and SMR methods during surgery. Surgical positions were divided into four groups: supine position (without head rotation), supine position with head rotation (HR supine position) (e.g., position for the pterional approach), lateral position (including park-bench), and prone position. Ten patients were scheduled for placement in each surgical position. Skin distortion was evaluated based on the difference in distance errors between the AIR and SMR methods.

**Figure 1:**
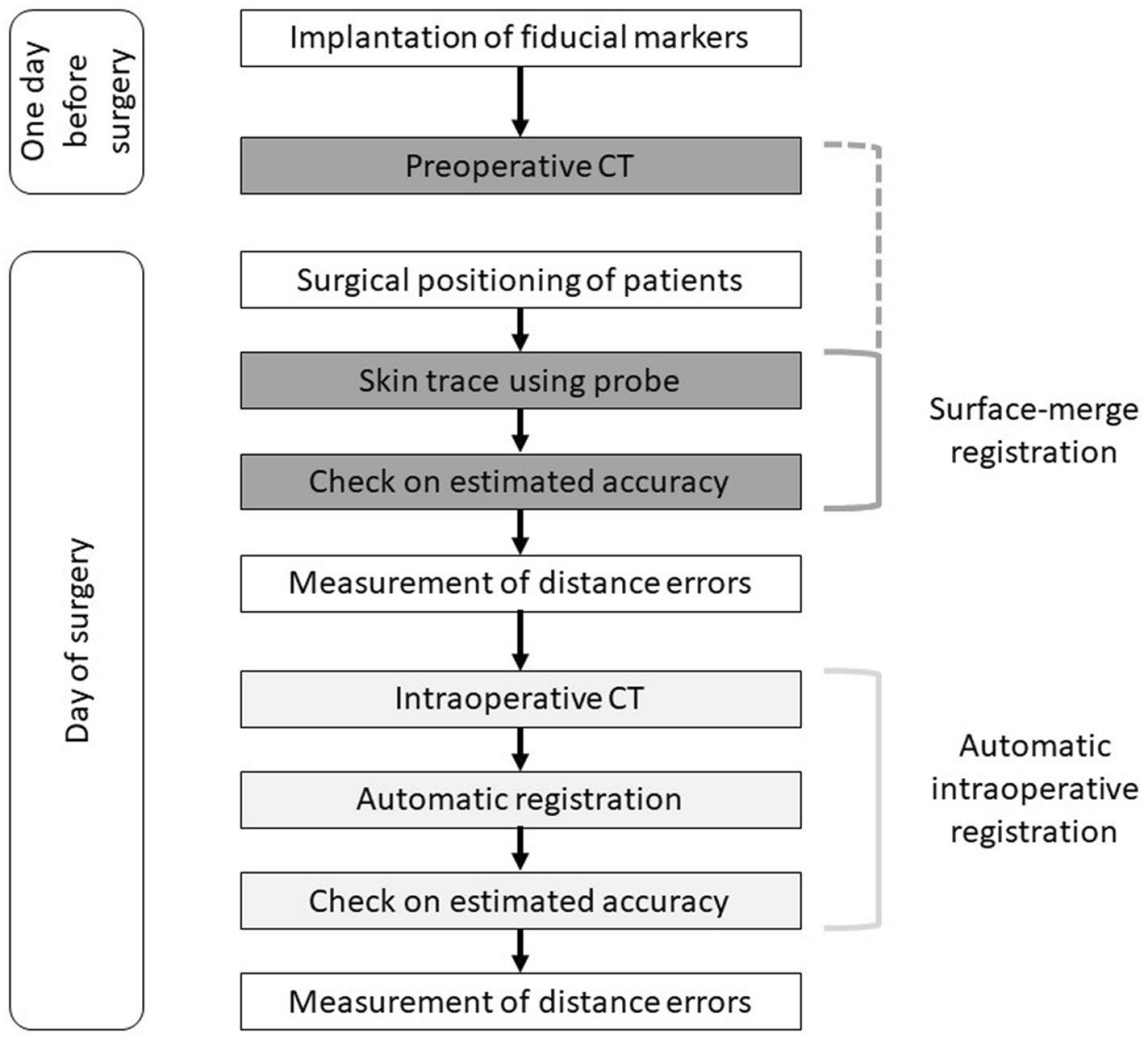
Workflow chart of registration method and distance error measurement.The surface-merge registration used a preoperative computed tomography for a reference image (dark gray). The skin of the patient’s head was traced using a navigation probe. The automatic intraoperative registration used an intraoperative computed tomography for a reference image (light gray). Distance errors of fiducial markers were measured after each registration.

**Figure 2:**
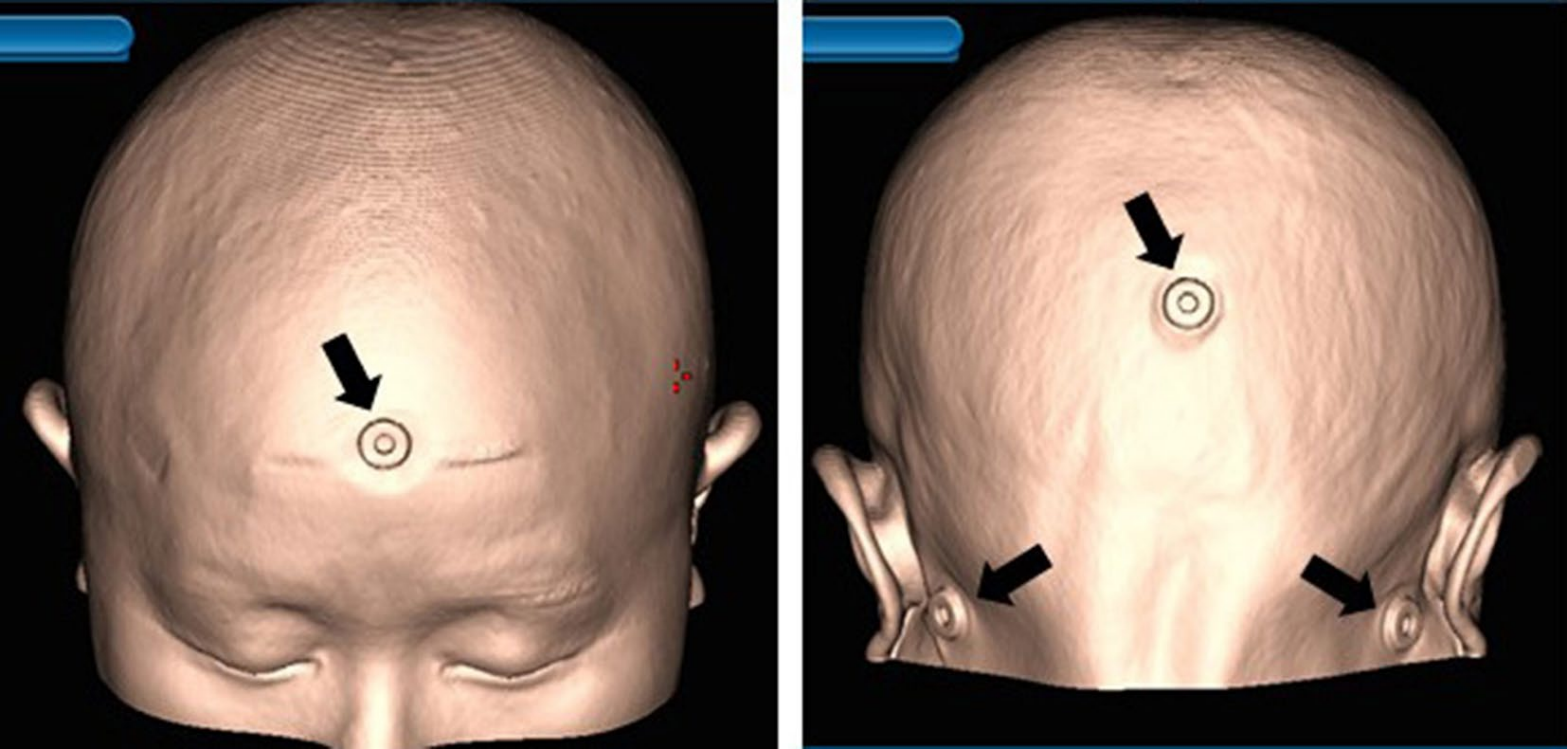
Fiducial markers were attached to the head as landmarks for the front, the back, the right, and left. A three-dimensional reconstruction using computed tomography shows that four fiducial markers (arrows) were implanted in the patient’s forehead (nasion), occipital region (inion), and bilateral retroauricle regions (mastoid process).

Figure 1 shows a work flow of registration of the navigation system and measurement of the distance errors. After obtaining an informed consent, four fiducial markers were implanted in the forehead (nasion), occipital region (inion), and bilateral retroauricular regions (mastoid process) (Fig. 2) one day before surgery. The markers on the forehead and occipital protuberance were placed in the midline. The markers on the mastoid process were symmetrically placed. These markers were landmarks of the anterior (forehead), right (supine and prone positions), ipsilateral to the operative field (other positions), left (supine and prone positions), contralateral to the operative field (other positions), and posterior (nasion) points. An influence of skin distortion on the whole head was investigated to measure distance errors of these four markers. The hair was shaved in the occipital region, where the fiducial marker was placed, to prevent slipping of the marker. Preoperative CT was performed to obtain reference images for the SMR method, and the fiducial markers were retained until surgery. The contours of the fiducial markers were also marked on the skin, to allow reattachment in the event that the markers fell off before surgery.

**Figure 3:**
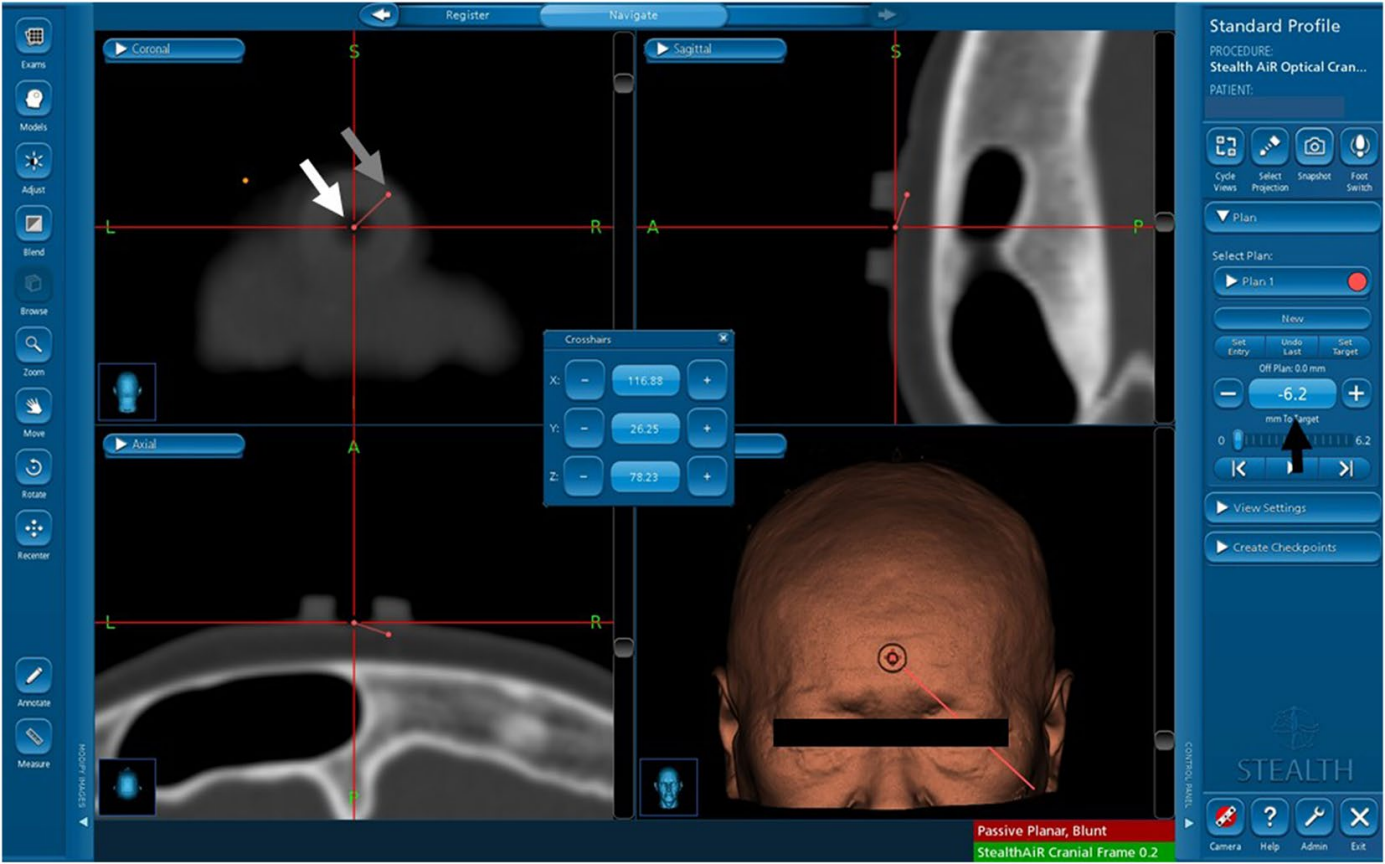
Using the surgical plan with the navigation system, the center of the fiducial marker on an image was set as the entry point (white arrow) and the center of the actual fiducial marker on a patient was set as the target point (gray arrow). The distance between the entry and target points was measured as the distance error of the navigation system (black arrow).

A Stealth Station S7 navigation system (Medtronic, Memphis, TN, USA) was used for surgical navigation. After patients were placed in the surgical position, registration of the navigation system was initially performed using the SMR method. The navigation system was manually registered with a trace of the surface anatomy, which included the nasal root and forehead (SMR method). Skin tracing is usually performed by neurosurgical residents in accordance with the registration instructions for the navigation system. Operators routinely checked the estimated accuracy of the navigation system using the nose, orbits, ears, and scalp surface in the operative field after the registration. The surgical plan featured in the navigation system to create target trajectories was used to measure the distances between the markers on the images in the navigation system and the actual markers. In the surgical plan, the center of the fiducial marker on an image was set as the entry point and the center of the actual fiducial marker on the patient was set as the target point (Fig. 3). The distance between the entry and target points is used to measure the distance errors of the navigation system. The xyz-components of the distance errors were also measured using the distances of the xyz coordinates between the entry and target points (Fig. 3).

After performing SMR, intraoperative images were obtained using a CT scanner (SOMATOM Definition AS, SIEMENS, Erlangen, Germany). The scanned images were reconstructed with reference to the orbitomeatal line and transferred to the navigation system through the local area network system, which was automatically registered using the AIR method. Subsequently, the distances between the markers on the images and on the patients were measured using the same procedure as the SMR method.

The xyz-coordinates of the entry and target points were analyzed to evaluate the direction of the distance error. The x-coordinate indicated the left–right axis (plus, left; minus, right); the y-coordinate indicated the perpendicular (dorsoventral) axis (plus, dorsal; minus, ventral); and the z-coordinate showed the craniocaudal axis (plus, cranial; minus, caudal). These coordinate directions were defined against the head in the supine position, irrespective of the surgical position, because intraoperative CT was reconstructed in the supine position. The differences between the entry and target points were calculated by subtracting the coordinates of the entry points (imaging marker position) from those of the target points (actual marker position). This difference in coordinates indicated each component of the distance error in the xyz-direction.

We analyzed the distance errors of the navigation system for each surgical position. Box plots show the median values, and 25th, and 75th quartiles. The whisker plot shows the maximum and minimum values. The “X” shows the average value. Comparisons of distance errors between the SMR and AIR methods were performed using the Wilcoxon signed-rank test. The xyz-coordinate values of distance errors in each marker were compared using analysis of variance (ANOVA) with a post hoc Tukey–Kramer test. Statistical significance was set at *p *< 0.05.

## Results

Fifty-five patients were enrolled between February 2019 and December 2020. The patient demographics are shown in Table 1. Thirteen and 17 patients were placed in the supine and head rotation (HR) supine positions, respectively. Fourteen and 11 patients were placed in the lateral and prone positions, respectively. One patient with recurrent hemifacial spasm caused by the dolichoectatic vertebral artery was included because surgical navigation was required.

**Table 1:** Patient characteristics.

	Surgical positions				
	All patients (n = 55)	Supine (n = 13)	Supine with head rotation (n = 17)	Lateral (n = 14)	Prone (n = 11)
Age (median age)(range)	69 years (19–93)	71 years (19–87)	69 years (33–81)	70 years (35–82)	68 years (51–93)
Sex, Male (%)/Female (%)	25 (45.5)/30 (54.5)	5 (38.5)/8 (61.5)	6 (35.3)/11 (64.7)	6 (42.9)/8 (57.1)	8 (72.7)/3(27.3)
Tumor (%)					
Meningioma	19 (34.5)	4 (30.8)	6 (35.3)	6 (42.9)	3 (27.3)
High grade glioma	9 (16.4)	1 (7.7)	5 (29.4)	1 (5.9)	2 (18.2)
Metastatic brain tumor	7 (13.0)	1 (7.7)	2 (11.8)	1 (5.9)	3 (27.3)
PCNSL	5 (12.7)	2 (15.4)	2 (11.8)		1 (9.1)
Vestibular schwannoma	3 (5.5)			3 (21.4)	
Rathke's cleft cyst	2 (3.6)	2 (15.4)			
Epidermoid	2 (3.6)		1 (5.9)	1 (5.9)	
Hemangiopericytoma	1 (1.8)				1 (14.3)
Craniopharyngioma	1 (1.8)	1 (7.7)			
Chordoma	1 (1.8)	1 (7.7)			
Hemangioblastoma	1 (1.8)				1 (9.1)
Dermoid cyst	1 (1.8)		1 (5.9)		
Cavernous malformation	1 (1.8)			1 (5.9)	
Arachnoid cyst	1 (1.8)	1 (7.7)			
Hemifacial spasm	1 (1.8)			1 (5.9)	

*PCNSL* Primary central nervous system lymphoma.

### Distance error of fiducial markers between imaging space and patient space

**Figure 4:**
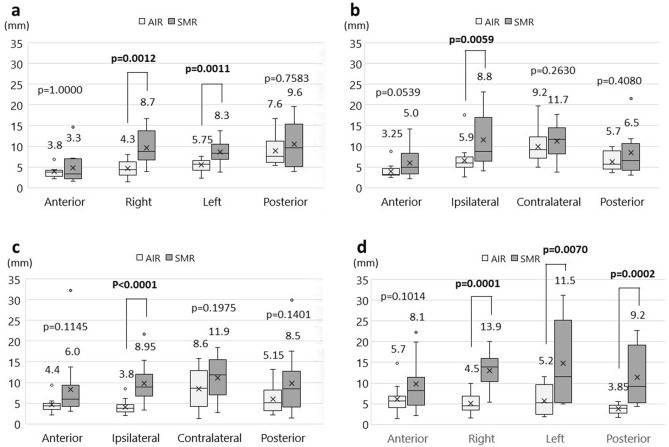
Distance errors of fiducial markers in each surgical position. (**a)** supine position (without head rotation); (**b)** supine position with head rotation; (**c)** lateral position; (**d)** prone position. Box plots show the median values with 25th and 75th quartiles. The whisker plots show the maximum and minimum values. The “X” shows the average value.

Distance errors of fiducial markers between reference images and the actual location of patients in each surgical position are shown in Fig. 4. In the supine position, the distance error in the anterior fiducial marker was the smallest among all the markers in the SMR and AIR methods (Fig. 4a). The median distance errors of the anterior and posterior markers were 3.3 mm and 9.6 mm in the SMR method and 3.8 mm and 7.6 mm in the AIR method, respectively. No statistically significant differences were observed in these markers between the SMR and AIR methods. Conversely, the distance errors of the right and left markers were significantly smaller in the AIR method than in the SMR method (right marker, 4.3 mm and 8.7 mm, *p *= 0.0012; the left marker, 5.75 mm, and 8.3 mm, *p *= 0.0011) (Fig. 4a). In the HR supine position, the median distance error in the anterior marker was also the smallest among all the markers in the SMR and AIR methods (Fig. 4b). The distance error of the marker ipsilateral to the surgical side was significantly smaller in the AIR method than in the SMR method (5.9 mm vs. 8.8 mm; *p *= 0.0059) (Fig. 4b). In the lateral position, the distance error of the anterior marker was the smallest among all markers in the SMR method, but not in the AIR method (Fig. 4c). The distance error of the ipsilateral marker was significantly smaller in the AIR method than in the SMR method (3.8 mm vs. 8.95 mm; *p *< 0.0001) (Fig. 4c). In the prone position, the distance error of the anterior marker also showed the smallest value among all the markers in the SMR method (Fig. 4d). In contrast, the distance error of the posterior marker was the smallest among all markers in the AIR method. The distance errors of the right, left, and posterior markers were significantly smaller in the AIR method than in the SMR method (right, *p *= 0.0001; left, *p *= 0.0070; posterior, *p *= 0.0002) (Fig. 4d).

### Xyz direction of distance errors in each fiducial marker

**Figure 5:**
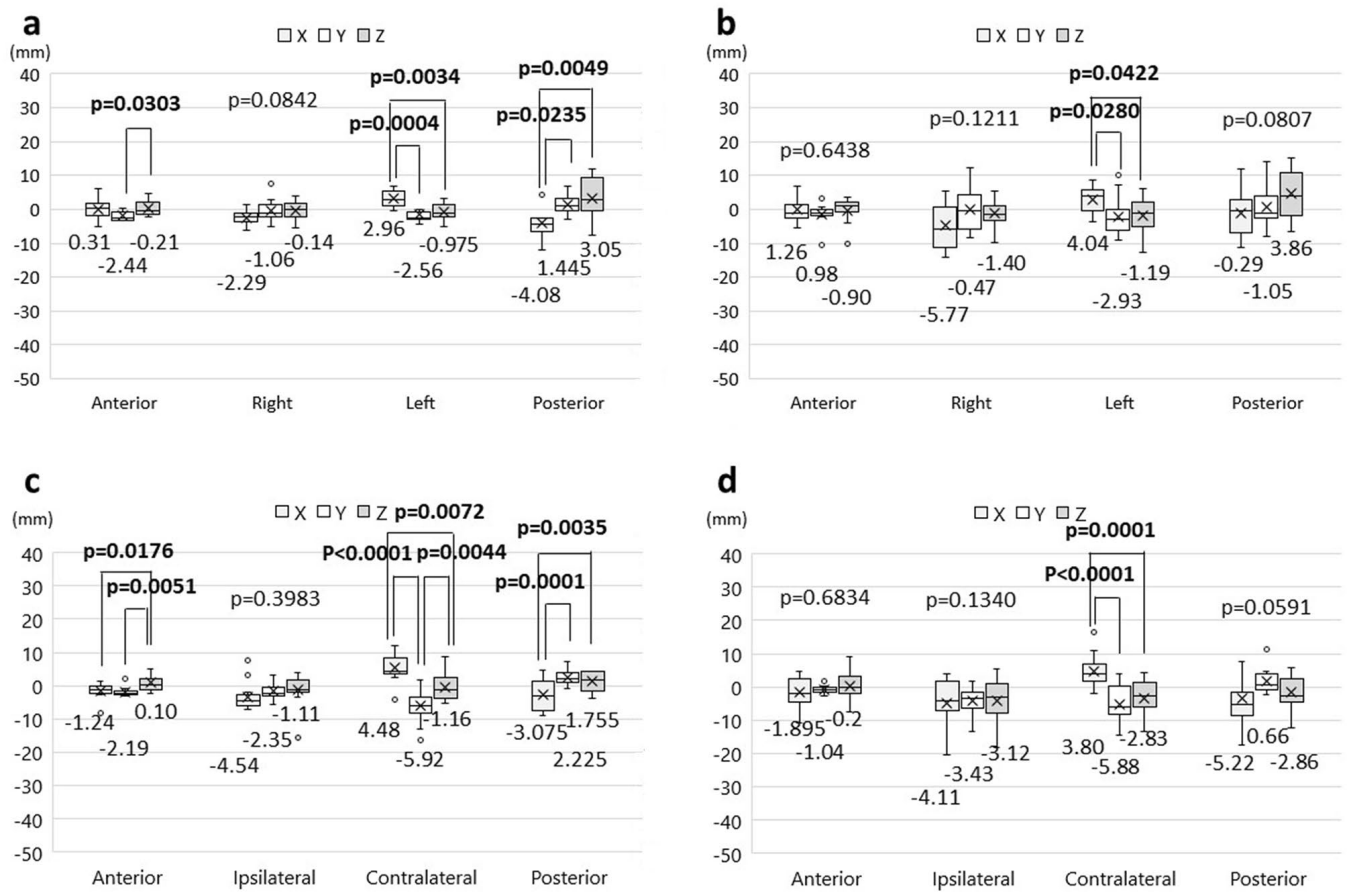
The direction of the distance errors for each fiducial marker in the supine position without and with head rotation. Box plots show the values of the distance errors in the xyz-components of each marker. (**a)** automatic intraoperative registration in the supine position; (**b)** surface-merge registration in the supine position; (**c**) automatic intraoperative registration in the supine position with head rotation; (**d**) surface-merge registration in the supine position with head rotation.

Table 2 shows the summary of distance errors and xyz directions of distance errors. In the supine position, there were statistically significant differences in the distance errors among the xyz-components in the anterior, left, and posterior markers using the AIR method (Fig. 5a). In a post hoc Tukey–Kramer analysis, statistically significant differences were seen between the y- and z-component in the anterior marker (*p *= 0.0303), the x- and y-component, and x- and z-component in the left marker (x vs. y, *p *= 0.0004; x vs. z, *p *= 0.0034), and the x- and y-component, and x- and z-component in the posterior marker (x vs. y, *p *= 0.0235; x vs. z, *p *= 0.0049). Regarding the SMR method, a significant difference was observed in the left marker (Fig. 5b). The post hoc analysis revealed significant differences between the x- and y-component, and the x- and z-component (x vs. y, *p *= 0.0280; x vs. z, *p *= 0.0422). In the HR supine position, ANOVA showed statistically significant differences in the anterior, marker contralateral to the surgical side, and posterior markers using the AIR method (Fig. 5c). In the post hoc analysis, there were significant difference between x- and z-component, and y- and z-component in the anterior marker (x vs. z, *p *= 0.0176; y vs. z, *p *= 0.0051), among all components in the contralateral marker (x vs. y, *p *< 0.0001; x vs. z, *p *= 0.0072; y vs. z, *p *= 0.0044), and between the x- and y-component, and x- and z-component in the posterior marker (x vs. y, *p *= 0.0001; x vs. z, *p *= 0.0035). In the SMR method, the xyz-components showed significant differences in the contralateral marker (Fig. 5d). The post hoc analysis revealed significant differences in the same pattern as the supine position (x vs. y, *p *< 0.0001; x vs. z, *p *= 0.0001).

**Table 2:** Distance error of fiducial markers between imaging space and patient space.

	Anterior marker (mm)				Right/ ipsilateral marker (mm)				Left/ contralateral marker (mm)				Posterior marker (mm)			
		Coordinate				Coordinate				Coordinate				Coordinate		
	DE	x	y	z	DE	x	y	z	DE	x	y	z	DE	x	y	z
Supine position																
AIR	3.3	0.31	− 2.44	− 0.21	4.3	− 2.29	− 1.06	− 0.14	5.75	2.96	− 2.56	− 0.975	7.6	− 4.08	1.445	3.05
SMR	3.8	1.26	0.98	− 0.90	8.7	− 5.77	− 0.47	− 1.40	8.3	4.04	− 2.93	− 1.19	9.6	− 0.29	− 1.05	3.86
HR supine position																
AIR	3.25	− 1.24	− 2.19	0.10	5.9	− 4.54	− 2.35	− 1.11	9.2	4.48	− 5.92	− 1.16	5.7	− 3.075	2.225	1.755
SMR	5.0	− 1.895	− 1.04	− 0.2	8.8	− 4.11	− 3.43	− 3.12	11.7	3.80	− 5.88	− 2.83	6.5	− 5.22	0.66	− 2.86
Lateral position																
AIR	4.4	− 1.16	− 1.785	2.535	3.8	− 1.715	1.805	− 0.785	8.6	2.605	3.095	1.50	5.15	− 0.64	3.18	2.095
SMR	6.0	− 1.32	− 0.71	− 0.40	8.95	− 2.71	− 0.38	0.76	11.9	1.22	8.11	− 2.20	8.5	− 2.75	2.38	− 0.01
Prone position																
AIR	5.7	1.38	− 1.43	3.72	4.5	− 0.54	0.95	0.54	5.2	2.26	2.245	0.865	3.85	− 1.22	2.075	− 0.12
SMR	8.1	3.08	− 0.74	4.10	13.9	− 0.46	10.38	− 0.44	13.9	0.84	9.11	2.50	9.2	− 0.86	2.55	5.38

*AIR* Automatic intraoperative registration, *DE* Distance error, *SMR* Surface-merge registration.

**Figure 6:**
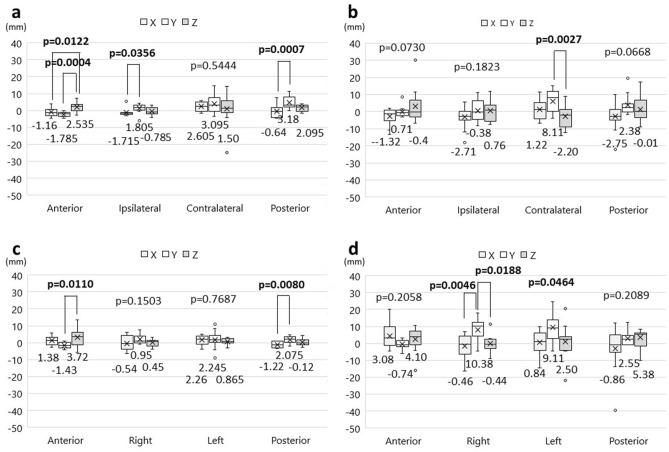
The direction of the distance errors for each fiducial marker in the lateral and prone positions. Box plots show the values of the distance errors in the xyz-components of each marker. (**a**), automatic intraoperative registration in the lateral position; (**b**) surface-merge registration in the lateral position; (**c**) automatic intraoperative registration in the prone position; (**d**) surface-merge registration in the prone position.

With regard to the lateral position, ANOVA revealed statistical differences among the xyz-components in the anterior, ipsilateral, and posterior markers registered by the AIR method (Fig. 6a). Significant differences were shown between x- and z-component, and y- and z-component in the anterior marker (x vs. z, *p *= 0.0122; y vs. z, *p *= 0.0004), between x- and y-component in the ipsilateral marker (*p *= 0.0356), and between x- and y-component in the posterior marker (*p *= 0.0007). In the SMR method, a significant difference was observed in the contralateral marker (Fig. 6b). The post hoc analysis revealed significant differences between the y- and z-component (*p *= 0.0027). In the prone position, the variance among xyz-components showed significant differences in the anterior and posterior markers registered by the AIR method (Fig. 6c). Using the post hoc paired test, significant differences were shown between y- and z-component in the anterior marker (*p *= 0.0110), and between x- and y-component in the posterior marker (*p *= 0.0080) (Fig. 6d). In the SMR method, ANOVA demonstrated significant differences between the right and left markers (Fig. 6d). The post hoc test showed significant differences between x- and y-component, and y- and z-component in the right marker only (x vs. y, *p *= 0.0046; y vs. z, *p *= 0.0188).

## Discussion

Needless to say, accuracy is a crucial factor in a navigation system. Registration is an important procedure for ensuring the accuracy of navigation systems. A new registration method has emerged with the development of surgical technology over time, and has been validated. Therefore, the accuracy of navigation systems has been reported in various studies over several decades. The errors in the navigation system were within 1–2 mm using the phantom model^4,6,7^, which is sufficient for clinical use. However, these errors did not include the skin distortion caused by the surgical position and skull pins of the head clamp. Therefore, studies evaluating the accuracy of the navigation system in clinical settings have also been reported^5,7–13^. There are several types of errors during registration in the navigation system : (1) fiducial location error, (2) fiducial registration error, and (3) target registration error (TRE)^14^. TRE is the most significant registration error for clinicians and has been investigated in surgical interventions. However, it was difficult to measure the exact TRE using anatomical landmarks or brain lesions as a target; navigation probe-indicated position during surgery could not be precisely evaluated in accordance with the image’s target position setting. In such cases, therefore, TRE had to be estimated^7–10^. Golfinos et al. determined the utility of the navigation system for locating lesions using the surgeon’s questionnaires (“yes” or “no”)^8^. Muacevic et al. measured the distance errors of the deep-seated lesions, between the probe tip position on the navigation monitor and it’s actual position^7^. However, how the actual probe position or the target lesion was defined on the navigation monitor was not described. These studies showed that TRE was mostly within 5 mm. To evaluate TRE accurately, Mongen et al. used a fiducial marker as a substitute for a target structure as with the present study^5^. The TREs were 2.49 mm for point-based registration and 5.35 mm for surface matching. Mitsui et al. evaluated skin shift due to surgical position and head clamp, and compared intraoperative magnetic resonance image (MRI) with preoperative MRI^15^. The skin shift was 5.04 ± 2.42 mm in supine position and 5.98 ± 2.94 mm in prone position. The Nimsky group reported that the TREs were within 1 mm using intraoperative images for reference^11–13^.

This study aimed to evaluate the clinical accuracy of a navigation system using AIR and SMR methods in various surgical positions. Moreover, we evaluated the influence of skin distortion on the surgical field and the whole head. Theoretically, the navigation system distance error using the AIR method should not include errors due to skin distortion caused by differences in the patient positions. The median distance errors of operative fields (e.g. the anterior in supine position, the posterior in prone position) were the smallest with a range of 3.25–4.5 mm among the other regions in the AIR method. The farther the marker was from the operative field, the larger the distance error, particularly in the marker on the contralateral side of the operative field (median distance error, 7.6–9.2 mm). This could be due to the probe pointing. It was not easy to detect navigation probe-optical markers by the navigation camera when the contralateral marker was pointed out. In such situations, an excessive angulation against the marker to be pointed by the probe is required, probably causing the skin distortion. In the SMR method, the distance error was the smallest (3.3–8.1 mm) in the anterior region irrespective of the surgical position. Registration instructions for the navigation system were recommended to trace the nasal root, forehead, and frontal skin for the SMR method because tracing these anatomical landmarks contributed to the accurate registration of the navigation system. Consequently, the surface matching could deviate to the anterior side regardless of the surgical position. In other words, the SMR method was suitable for the supine position but not for the other surgical positions. Therefore, fiducial markers should be implanted in and around surgical field when the point-based registration is used for the navigation system. Dho et al. compared navigation accuracy using preoperative MRI scans as reference images between the supine and prone positions, in patients who underwent surgery in the prone position^16^. The anatomical points were matched to images with a higher rate during surgeries that used reference images taken in the prone position (60/64, 93.8%) than in the supine position (4/64, 6.2%). The authors also reported that the average distance of distortion between supine and prone MRI was 6.3 mm. Although it could potentially improve the accuracy of the navigation system for the SMR method to perform preoperative MRI in the same position as the surgical position, it is not practical. Alternatively, surface-matching should be performed in regions close to the operative field. However, the clinical accuracy of the navigation system when surface-matching is performed in a region different from the forehead is unknown.

**Figure 7:**
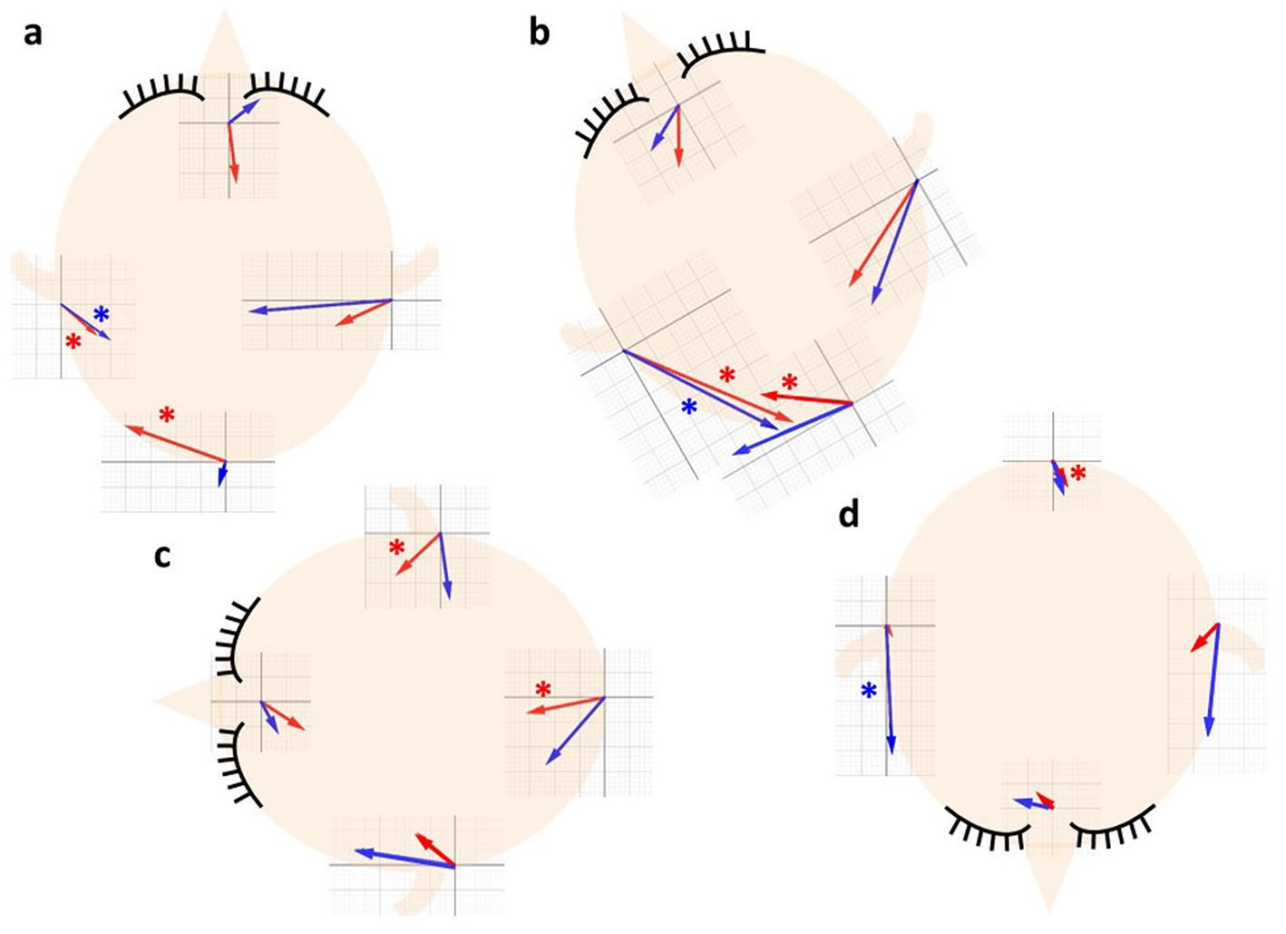
A schematic diagram of x–y vectors of the distance errors. An illustration of the head showing the imaging space. The vectors indicate the direction of the deviation from the imaging point to the patient point in the AIR method (red arrows) and the SMR method (blue arrows), respectively. Asterisks show the significant differences between x- and y-coordinates using an analysis of variance with a post hoc Turkey–Kramer test. (**a**) the supine position; (**b**) the head-rotated supine position; (**c**) the lateral position; (**d**) the prone position.

We hypothesized that the distance errors between the AIR and SMR methods could indicate errors due to skin distortion caused by differences in patient positions. In comparison of the AIR and SMR methods, the AIR method was significantly more accurate for the right and left markers in the supine and prone positions and for the ipsilateral markers in the HR supine and lateral positions than the SMR method. Moreover, in the prone position, the AIR method had a significantly high accuracy in the posterior marker than the SMR method. Therefore, in the lateral position, the distance error in SMR method should be considered, even in the operative field; the ipsilateral marker, which was close to the operative field, showed a large distance error. The xyz-coordinates of the distance errors were evaluated to elucidate the direction of the distance errors. Generally, the AIR method had more markers with significant coordinate variance than the SMR method. The SMR method could include more errors than the AIR method, cancelling each other out. Figure 7 shows a schematic diagram of the x–y vectors to make the direction clearly understandable. The vector indicates the deviation from the imaging point to the patient point. In the AIR method, the patient’s point was shifted downward from the imaging point in the anterior and bilateral markers. Conversely, the patient’s point the bottom marker shifted upwards. These shifts indicate the direction of pushing the scalp using a navigation probe. The vectors in the SMR method were directed more downward than those in the AIR method were. This direction can be combined with the direction of skin distortion by probe pointing and gravity. In summary, probe pointing may be a major cause of distance errors in the AIR method. Conversely, gravity-induced scalp distortion could be related to distance errors in the SMR method in addition to probe pointing.

This study had some limitations. Primarily, we used fiducial markers as substitutes for target lesions. The distance error of the fiducial markers showed the registration errors for the surface of the head. The distance errors in the deep brain structures could not be examined in this study. Even a 6° head-down position caused brain shift and volumetric changes in the brain, blood, and cerebrospinal fluid on MRI^17^. Therefore, gravity-induced brain distortion could not be evaluated from outside the cranium. Another limitation of our study, was that our distance errors included human errors, such as pointing error. Human errors obscure the true influence of scalp distortions on registration errors. However, the distance errors in this study were observed in the real world. The distance errors of the markers far from the operative field were impractical during the corresponding surgery. However, this study elucidated the influence of scalp distortion due to the surgical positioning of the whole head. Based on our results, surface-matching should be performed close to the operative field, and not all over the head, to improve the accuracy of the navigation system in the SMR method. Position of fiducial markers and reference array could cause inaccuracy of the navigation system. Fiducial markers attached the skin could be movable. Therefore, markers should be implanted on the skin where the underlying tissue was limited^1^. The skin over the mastoid, the frontal and parietal bones, and the forehead was less movable than other regions^18^. The closer a reference array was to the operative field, the more accurate the navigation was. Therefore, reference array was better to be set as close as possible to the patient’s head^19^. Using intraoperative CT for a reference image, a reference array had to be set very close to the patient’s head in order to include patient’s head and a reference array in the scan range. The small sample size is also a study limitation. It was difficult to statistically estimate an appropriate sample size because there were no data on the distance errors of the SMR and AIR methods. A large sample size could contribute to elucidating the differences between the two methods. However, the number of patients included in this study should be minimized. It was not necessary for patients to register the navigation system twice, and double registrations extended the duration of anesthesia and surgery. A sample size of 10 was selected as a practicable number considering the number of brain tumor surgeries requiring the navigation system in our institute.

In conclusion, the AIR method had smaller distance errors than the SMR method and the SMR method was more accurate in the frontal region, where surface matching was performed during the registration, than in the other regions irrespective of the surgical position. The AIR method was accurate and recommended for registration in all surgical positions if intraoperative imaging was available. Skin distortion frequently occurs in the lateral regions of the head and could be caused by differences between patient positions during preoperative imaging and surgery. Therefore, the SMR method was only recommended for the supine position, but not for the lateral and prone positions.

## Data Availability

The datasets generated and/or analyzed during the current study are available from the corresponding author upon reasonable request.
